# Polydopamine Functionalized Graphene Oxide as Membrane Nanofiller: Spectral and Structural Studies

**DOI:** 10.3390/membranes11020086

**Published:** 2021-01-27

**Authors:** Abedalkader Alkhouzaam, Hazim Qiblawey, Majeda Khraisheh

**Affiliations:** Department of Chemical Engineering, College of Engineering, Qatar University, P.O. Box 2713 Doha, Qatar; 200602139@student.qu.edu.qa (A.A.); m.khraisheh@qu.edu.qa (M.K.)

**Keywords:** graphene oxide, amine-functionalized GO, polydopamine, hydrophilic nanofiller, dispersibility, aminated GO

## Abstract

High-degree functionalization of graphene oxide (GO) nanoparticles (NPs) using polydopamine (PDA) was conducted to produce polydopamine functionalized graphene oxide nanoparticles (GO-PDA NPs). Aiming to explore their potential use as nanofiller in membrane separation processes, the spectral and structural properties of GO-PDA NPs were comprehensively analyzed. GO NPs were first prepared by the oxidation of graphite via a modified Hummers method. The obtained GO NPs were then functionalized with PDA using a GO:PDA ratio of 1:2 to obtain highly aminated GO NPs. The structural change was evaluated using XRD, FTIR-UATR, Raman spectroscopy, SEM and TEM. Several bands have emerged in the FTIR spectra of GO-PDA attributed to the amine groups of PDA confirming the high functionalization degree of GO NPs. Raman spectra and XRD patterns showed different crystalline structures and defects and higher interlayer spacing of GO-PDA. The change in elemental compositions was confirmed by XPS and CHNSO elemental analysis and showed an emerging N 1s core-level in the GO-PDA survey spectra corresponding to the amine groups of PDA. GO-PDA NPs showed better dispersibility in polar and nonpolar solvents expanding their potential utilization for different purposes. Furthermore, GO and GO-PDA-coated membranes were prepared via pressure-assisted self-assembly technique (PAS) using low concentrations of NPs (1 wt. %). Contact angle measurements showed excellent hydrophilic properties of GO-PDA with an average contact angle of (27.8°).

## 1. Introduction

Graphene and its based materials have been extensively researched recently owing to their unique and attractive properties. Graphene, which is extracted from natural graphite, is a two-dimensional material and is made of sp^2^ hybridized bonded carbon atoms arranged in a honeycomb structure [1]. One of the commonly explored graphene derivatives is graphene oxide (GO) that is usually produced from graphite sheets [2]. GO exhibits attractive optical, chemical, and electrical properties caused by the graphene skeleton and its oxygen content. The oxygenated functional groups located on the edges of GO sheets cause its hydrophilic properties and make the surface modifications easier to produce other graphene derivatives [1,3]. GO NPs are generally synthesized by the oxidation of graphite via the well-known Hummers method [2] and modified/improved Hummers methods that were developed later to improve the quality of GO NPs [4,5,6].

Interestingly, GO properties can be significantly tuned/enhanced by a successful functionalization for a wide range of applications. One of the efficient functionalization methods being done in this aspect is the amine functionalization of GO NPs. The amination of GO was investigated in several studies using different amines and was found to enhance the GO properties such as the hydrophilicity, dispersibility, conductivity, antibacterial properties, surface area, adsorption capacity, mechanical and thermal stability, and the antifouling properties of the materials in contact with water like membranes [7]. Aminated GO NPs were considered as a promising nanofiller in various membrane processes like reverse osmosis (RO), forward osmosis (FO), nanofiltration (NF) and ultrafiltration (UF). In a recent study, Zhang et al. [8] investigated the effect of p-aminophenol-functionalized GO embedding into thin-film nanocomposite RO membranes (TFN-RO) and reported a remarkable increase in water flux compared to bare membranes (by 24.5%) and high NaCl rejection of 99.7%. Further, TFN membranes incorporating the aminated GO exhibited higher antibacterial activity against *Escherichia coli* (96.78%) compared to those incorporating pristine GO (90.64%). In a similar study, Xue et al. [9] prepared TFN-NF membranes incorporating octadecylamine-functionalized GO (GO-ODA). The GO-ODA based membranes exhibited significant enhancement in the flux (2.5 times the pristine membrane) and excellent chlorine resistance while maintaining high rejection of sodium sulfate (98.4%). N-butylamine functionalized GO (GO-ButA) NPs were reported to have better dispersibility and stability in polymeric solutions and provide higher mechanical stability for polymeric composites [10,11]. Other applications of the aminated GO NPs include sensing applications [12], supercapacitors [13], catalysis [14], CO_2_ adsorption [15], and microwave adsorption [16]. Table 1 summarizes the recently reported amine-functionalized GO NPs and their applications.

In 2007, Lee et al. [20] found that dopamine could be self-polymerized in alkaline media to produce a hydrophilic thin polymeric film (polydopamine) that can be used for various coating applications. Since then, polydopamine (PDA) have been extensively explored for a wide range of application in material science. Further, PDA is considered an adaptable platform for further functionalization with the desired functional groups for different purposes because of the presence of abundant functional groups on its surface [21,22]. Because of such unique properties over other amines, PDA has been widely utilized for the functionalization/modification of several nanomaterials like multi-walled carbon nanotubes [23], silver NPs [24], SiO_2_ and TiO_2_ NPs [25,26]. The amination of GO NPs using PDA was reported in some studies for anticorrosion coatings purposes [27], electrocatalysis applications [28], and sensing applications [29], too. However, no enough investigations on the structural characteristics of GO-PDA have been reported yet. In this work, we report a high-degree functionalization of GO NPs with PDA by increasing the PDA: GO ratio (2:1) and the reaction time via a simple temperature-assisted reflux approach. A comprehensive analysis using different analytical techniques was conducted to investigate the chemical, structural, morphological, and thermal properties of the produced GO-PDA and to confirm the successful functionalization. These techniques include XPS, XRD, Raman spectroscopy, FTIR-UATR, CHNSO elemental analysis, SEM, and TGA. Furthermore, the hydrophilicity and dispersion properties of the GO-PDA NPs in different polar and nonpolar solvents were investigated to confirm its potential use as nanofiller in membrane technology with various membrane materials and for different purposes.

## 2. Materials and Methods

### 2.1. Materials

Natural graphite flakes (-10 mesh, 99.9%) were supplied by Alfa Aesar, Potsdam, Germany. KMnO_4_ (99%), H_2_SO_4_ (95%), and toluene (≥99.5%) were obtained from Fisher Scientific (Waltham, MA, USA). H_3_PO_4_ (99%), H_2_O_2_ (30%), HCl (35 to 38%), and hexane (≥98.5%) were purchased from BDH middle east, Dubai, UAE. Dopamine hydrochloride (DA), ethanol (≥99.8%), tris(hydroxymethyl)aminomethane (Tris), N,N-Dimethylacetamide (DMA, ≥99%), and 1-Methyl-2-pyrrolidinone (NMP, 99.5%) were purchased from Sigma Aldrich (St. Louis, MO, USA). dodecane (≥99%) and N,N-Dimethylformamide (DMF, ≥99.8%) were obtained from Honeywell (Charlotte, NC, USA). Polysulfone substrate (PS-30) with 20 K molecular weight cut-off was purchased from Sepro Membranes, Oceanside, CA, USA. The deionized water (DIW) was produced using the ELGA PURELAB Option water purification system, Lane End, UK. All materials were used as obtained without additional purification.

### 2.2. GO NPs Preparation

**Figure 1:**
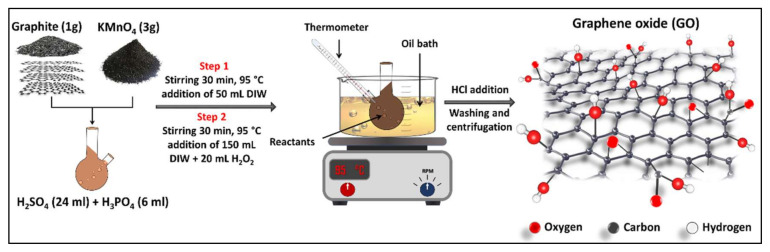
Schematic illustration of GO synthesis via the modified Hummers method.

GO NPs were prepared using a modified Hummers method described in earlier work [30]. In brief, 24 mL of H_2_SO_4_ and 6 mL of H_3_PO_4_ (4:1 volume ratio) were mixed and stirred in an ice bath for 5 min. Then 1 g of graphite and 3 g of KMnO_4_ were gradually added into the acid mixture under stirring condition. The reactants mixture was then placed in an oil bath at a temperature of 95 ± 2 °C for 30 min. 50 mL of DIW were then added to the reactants mixture at the same conditions for additional 30 min. The reactants mixture was then shifted to an ice bath and 20 mL of H_2_O_2_ and 150 mL of DIW were slowly added to the mixture to terminate the reaction. The resultant solution was then kept at room temperature to cool down, diluted and washed with 20% HCl solution and was then centrifuged at 7500 rpm for 20 min. The supernatant was then decanted, and the residuals were washed with DIW and centrifuged many times until the pH became around 6. Finally, the products were dried in an oven at 80 °C for about 48 h. Figure 1 illustrates the oxidation procedures of graphite to GO.

### 2.3. Functionalization of GO with Dopamine

**Figure 2:**
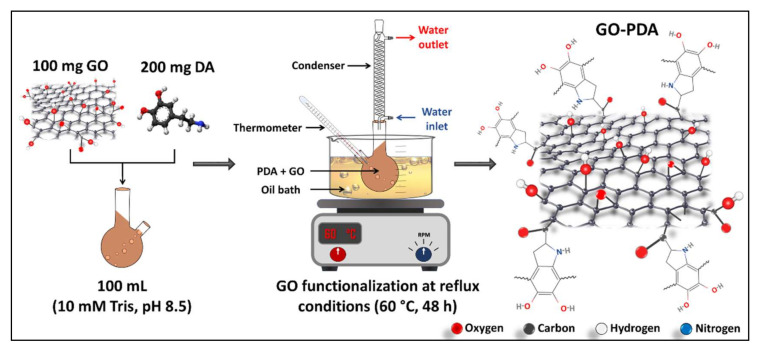
Illustration of the functionalization reaction of GO with polydopamine (PDA).

The amine functionalization of GO NPs with PDA was performed via the temperature-assisted reflux method [11]. Briefly, 100 mL of 10 mM Tris solution was prepared, and the pH was adjusted to 8.5 using HCl. 100 mg GO and 200 mg DA were dispersed in the Tris solution using a bath sonicator for 1 h. The GO-PDA suspension was stirred at 60 °C for 48 h in an oil bath under the reflux conditions. The functionalized NPs (GO-PDA) were then washed and extracted by the solvent evaporation approach. The resulted product was in the form of fine powders and was dried under vacuum at 80 °C overnight. Figure 2 illustrates the functionalization reaction of GO and the anticipated chemical structure of GO-PDA.

### 2.4. Characterization of GO and GO-PDA NPs

The produced NPs were characterized using various characterization techniques to confirm the functionalization and to explore its effects on GO properties. XPS measurements were conducted over a 0–1200 eV range on a Kratos AXIS Ultra DLD (Kratos Analytical Ltd, Manchester, UK) with Al-Kα source and X-ray power of 15 Kv and 20 mA. The elemental compositions of GO and GO-PDA were analyzed using FLASH 2000 elemental analyzer (Thermo Scientific™, Waltham, MA, USA). XRD measurements were carried out using EMPYREAN PANalytical diffractometer (Malvern Panalytical B.V., Eindhoven, Netherlands) equipped with a Cu-Kα radiation source (λ = 1.5406 Å). FTIR-UATR spectra were determined using FTIR Perkin Elmer 2000 in the range of 400–4000 cm^−1^ to study the surface functional groups of pristine GO and GO-PDA.

Raman spectra of the prepared NPs were obtained with DXR Raman Spectrometer operated with a 532 nm laser and a 10× objective (Thermo Scientific™). Moreover, the morphological structure of GO and GO-PDA NPs was investigated using SEM analysis that was conducted using the JEOL model JSM-6390LV. TGA analysis was conducted to assess the thermal stability of both samples using Pyris 6 TGA (PerkinElmer, Waltham, MA, USA) under nitrogen gas at a 10 °C/min heating rate and over a temperature range of 30–800 °C.

### 2.5. Dispersibility and Hydrophilicity Measurements

To investigate their dispersion properties, pristine GO and GO-PDA were dispersed in DIW and different organic solvents including, hexane, DMA, DMF, dodecane, toluene, and NMP. The dispersion tests were performed in an ultrasonic bath (SONREX DIGITEC DT 255 H, BANDELIN electronic, Berlin, Germany) for 2 h at room temperature and a concentration of 0.5 mg/mL.

**Figure 3:**
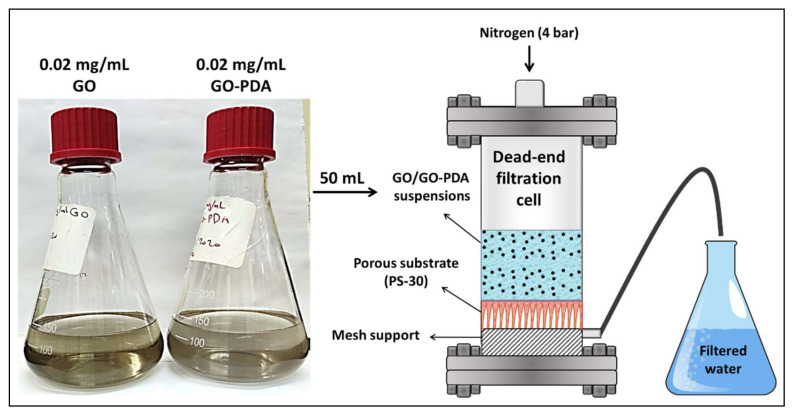
Illustration of the GO/GO-PDA assembly using the pressure-assisted self-assembly (PAS) technique.

To investigate the hydrophilicity properties of the pristine GO and GO-PDA, both samples were deposited on a PS-30 substrate using the pressurized assisted self-assembly (PAS) approach [31]. In brief, two stock dispersions containing 0.02 mg/mL of GO and GO-PDA NPs in DIW were first prepared using an ultrasonic bath sonicator for 2 h to ensure a good dispersion of the NPs (Figure 3). 50 mL of the stock solution was then transferred to a dead-end membrane cell (Sterlitech, Kent, WA, USA) with effective cross-sectional area of 14.6 cm^2^. The cell was then pressurized with 4 bar of nitrogen gas to force the water to pass through the substrate while GO/GO-PDA NPs are being assembled. The NPs composition in the obtained membranes is 1 wt. % with respect to the effective area of PS-30. The obtained membranes were then dried in an oven at 50 °C. Figure 3 illustrates the PAS technique for PS/GO and PS/GO-PDA membranes preparation. The hydrophilicity of the pristine and the coated membranes was then explored using the OCA15 Pro contact angle analyzer (DataPhysics, Stuttgart, Germany). The contact angle measurements were carried out at room temperature and using a DIW droplet of 2 µm at different points of each membrane sample (minimum of 10 points) and the average contact angle value was then calculated.

## 3. Results

### 3.1. Structural Properties of GO and GO-PDA

**Figure 4:**
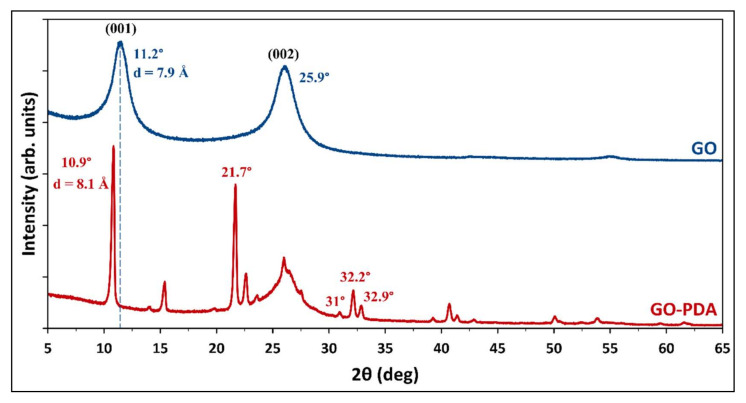
XRD patterns of GO and GO-PDA nanoparticles (NPs).

**Figure 5:**
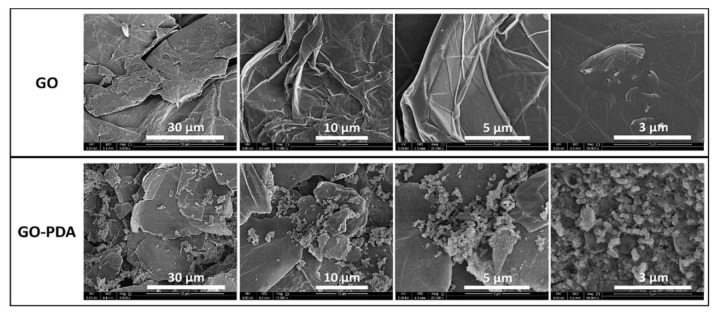
SEM images of GO and GO-PDA NPs.

**Figure 6:**
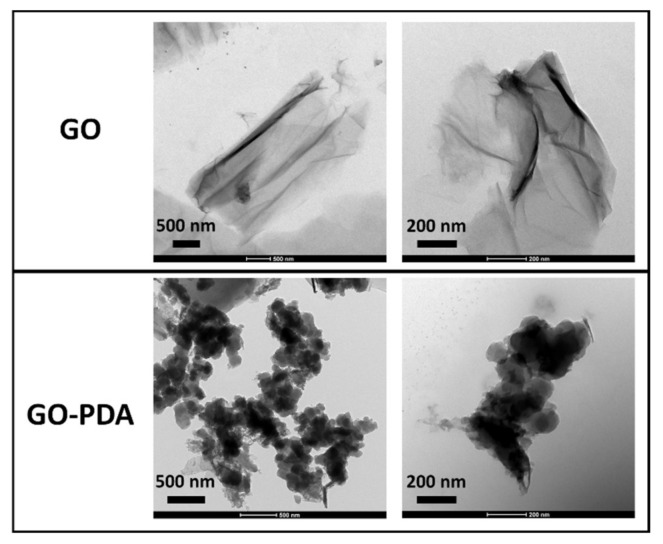
TEM images of GO and GO-PDA NPs.

The structural change represented by the XRD patterns of pristine GO and GO-PDA NPs is shown in Figure 4. The diffraction peak of the GO sample at 11.2° corresponds to the 001 plane of the hexagonal crystal structure of GO [4]; while the peak at 25.9° corresponds to the 002 plane which can be attributed to unoxidized graphite in the synthesized GO [32,33,34]. The incomplete oxidation of graphite results in the formation of graphite-GO mixture which is considered one of the main drawbacks of Hummers-based methods [6]. For GO-PDA, the 001 plane was shifted to 10.9° with the presence of several peaks confirming the structural change resulted from the functionalization reaction. A sharp diffraction peak has emerged in the GO-PDA patterns at 21.7° which is close to the graphite diffraction peak at 25.9° indicating a partial reduction of GO [35]. The diffraction peaks at 31°, 32.2° and 32.9° have been previously reported with PDA functionalized carbon nanotubes (CNT-PDA) and they were related to the PDA [36]. Further, the diffraction peaks around 41.4° and 42.9° were previously reported with rGO/PDA NPs [37]. The interlayer spacing of the samples (d-spacing) can be correlated to the oxygenated functional groups of GO. The interlayer spacing of the 001 plane (d_(001)_-spacing) was calculated by Bragg’s equation to be 7.9 Å for GO which is close to the values reported for GO synthesized by modified Hummers methods [4,33,38]. The d_(001)_-spacing of GO-PDA was found to be 8.1 Å indicating a slight expansion in the interlayer spacing. The expansion in the interlayer spacing confirms the formation of new oxygenic functional groups from the PDA between the GO layers. This can be also demonstrated in the SEM images of GO and GO-PDA presented in Figure 5. SEM images at different magnifications show a clear difference in the morphological characteristics resulted from the amination of GO. Images of the unfunctionalized GO NPs exhibit sharp, clear, and smoother flakes while the GO-PDA NPs exhibited rougher surface and irregular structure. SEM images at high magnifications show well distribution and attachment of PDA particles on the surface and between the GO sheets which lower the stacking level of GO layers and expands the interlayer spacing as demonstrated by XRD results. The TEM images in Figure 6 depict two distinct morphologies of GO and GO-PDA NPs. GO NPs exhibited wrinkled surface and highly transparent sheets which can be related to the lower stacking level of GO sheets. The high transparency and wrinkled surface indicate a high oxidation level of GO sheets [30,39]. In contrast, the TEM images show dense and opaque surface of the GO-PDA NPs indicating a successful grafting of PDA on the GO surface, which agrees with the results obtained by the SEM images.

**Figure 7:**
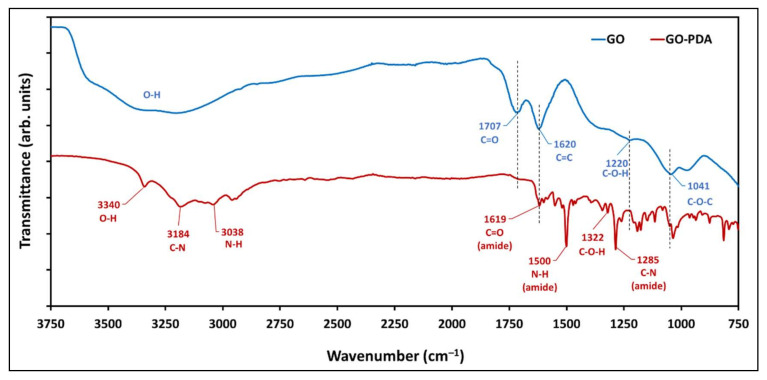
FTIR spectra of the pristine GO and GO-PDA NPs.

The FTIR spectra of the pristine GO and GO-PDA NPs are shown in Figure 7. The spectra of the prepared GO confirm the oxidation of graphite owing to the presence of several bands attributed to oxygen functionalization around ~1041, 1220, 1707 cm^−1^ corresponding to C–O–C epoxy stretching vibration, C–OH bending vibrations of hydroxyl groups, and the C=O stretching vibration of carbonyl groups on the edges of GO sheets, respectively. The band at ~1620 cm^−1^ corresponds to C=C skeletal vibration from unoxidized graphene [5,39] while the band at ~3300 cm^−1^ corresponds to the O–H stretching vibrations which is attributed to the water molecules intercalated between the GO sheets [6]. The successful functionalization of GO NPs can be confirmed by the emerging bands in the spectra of GO-PDA that are attributed to the amide functionality at ~1285, 1500, 1619, 3038, and 3184 cm^−1^. These bands have been previously reported in literature and were related to PDA [40,41,42]. Further, it was found that the C=O band at 1707 cm^−1^ was almost disappeared with GO-PDA demonstrating a partial reduction of GO NPs [28].

**Figure 8:**
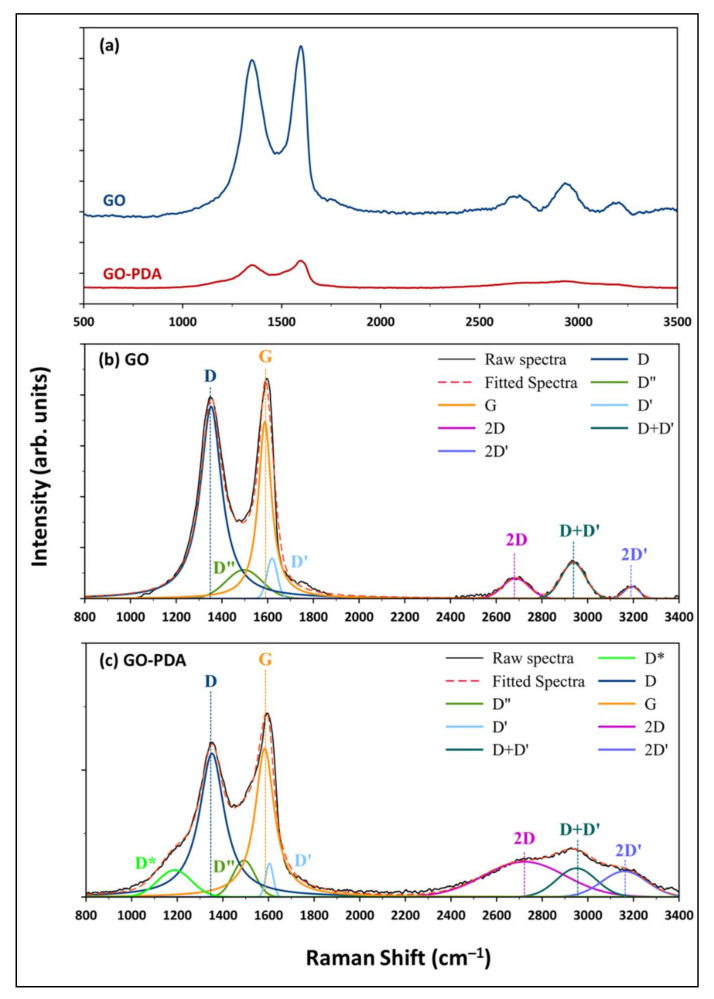
(**a**) Raman spectra of GO and GO-PDA and illustration of Raman spectra deconvolution and peaks fitting for (**b**) GO and (**c**) GO-PDA.

Raman spectroscopy is a crucial tool for analyzing GO and other graphene-based materials. A good analysis of the Raman spectra provide quantitative and qualitative information about the properties of GO like defects, the number of layers, and crystallite size [43]. Raman spectra of the synthesized GO and GO-PDA are shown in Figure 8a. The two distinctive bands for graphene-based materials, G and D, are existing in both spectra at ~1590 and ~1350 cm^−1^, respectively. It is well-known that the intensities ratio of these bands (*I_D_*/*I_G_*) in the first-order spectra is related to the crystallite size of GO NPs [44]. Several studies reported that the *I_D_*/*I_G_* ratio is inversely proportional to the sp^2^ crystallite size [44,45]. Consequently, the first-order spectra of both samples were deconvoluted and fitted into four peaks, D, D”, G, and D’ at ~1352, 1495, 1585, and 1610 cm^−1^, respectively. A fifth peak (D*) emerged in the spectra of GO-PDA at ~1188 cm^−1^ which was earlier reported and attributed to disordered graphitic lattices [46,47]. The *I_D_*/*I_G_* ratio was then estimated from the fitted spectra. Figure 8b,c) depicts the spectra deconvolution and peak fitting of GO and GO-PDA, respectively. From the estimated intensities (*I_D_* and *I_G_*), the crystallite size *(L_a_*, nm) for both samples was then calculated using the Tuinstra−Koenig model [48]:

$$L_{a}=\left(2.4\times 10^{-10}\right)\lambda^{4}{\left(I_{D}/I_{G}\right)}^{-1}$$

where *λ* is the laser wavelength (nm). The parameters of G and D bands and the evaluated crystallite sizes *L_a_* of GO and GO-PDA NPs are presented in Table 2. The crystallite sizes estimated from the *I_D_*/*I_G_* ratios were found to be 10.9 and 15.2 nm for the pristine GO and GO-PDA, respectively. Further, the *I_D_*/*I_G_* ratio of GO-PDA (1.3) was found to be lower than this of the pristine GO (1.8) indicating lower oxygen content of GO-PDA [39,49]. This is in a good agreement with the results obtained from XRD and FTIR analyses. The complete bands parameters are listed in Table S1 in the supplementary information. Second-order bands around (~2500–3200 cm^−1^) are also presented in the spectra of both samples which is attributed to second-order phonon processes [43]. The second-order spectra were deconvoluted into three peaks, 2D, D+D’, and 2D’, as depicted in Figure 8. Bands parameters estimated from the deconvolution and fitting of the second-order spectra are listed in Table S2 of the supplementary information. The second-order bands are associated with the quality of GO [50]. The positions of the 2D and D+D’ peaks were reported to be good estimators of graphitization degree, represented by Csp^2^ content, in GO NPs [47]. It was demonstrated in our previous work [30] that the correlations reported by López-Díaz et al. [47] don’t always provide accurate values of the Csp^2^ content especially when other elements like nitrogen are presented in the sample. However, it can be said that the positions of 2D and D+D’ shifts to higher wavenumbers with samples having lower oxygen contents. Therefore, the slight shift of 2D and D+D’ positions in the GO-PDA spectra could be attributed to the lower oxygen content of GO-PDA due to the partial reduction resulted from the functionalization reaction.

### 3.2. Compositional Properties of GO and GO-PDA

**Figure 9:**
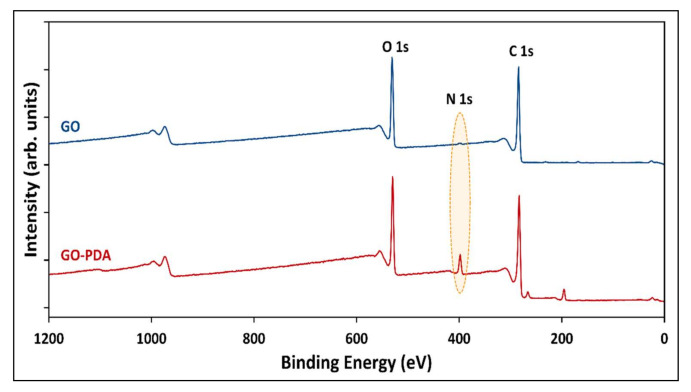
XPS survey spectra of GO and GO-PDA.

**Figure 10:**
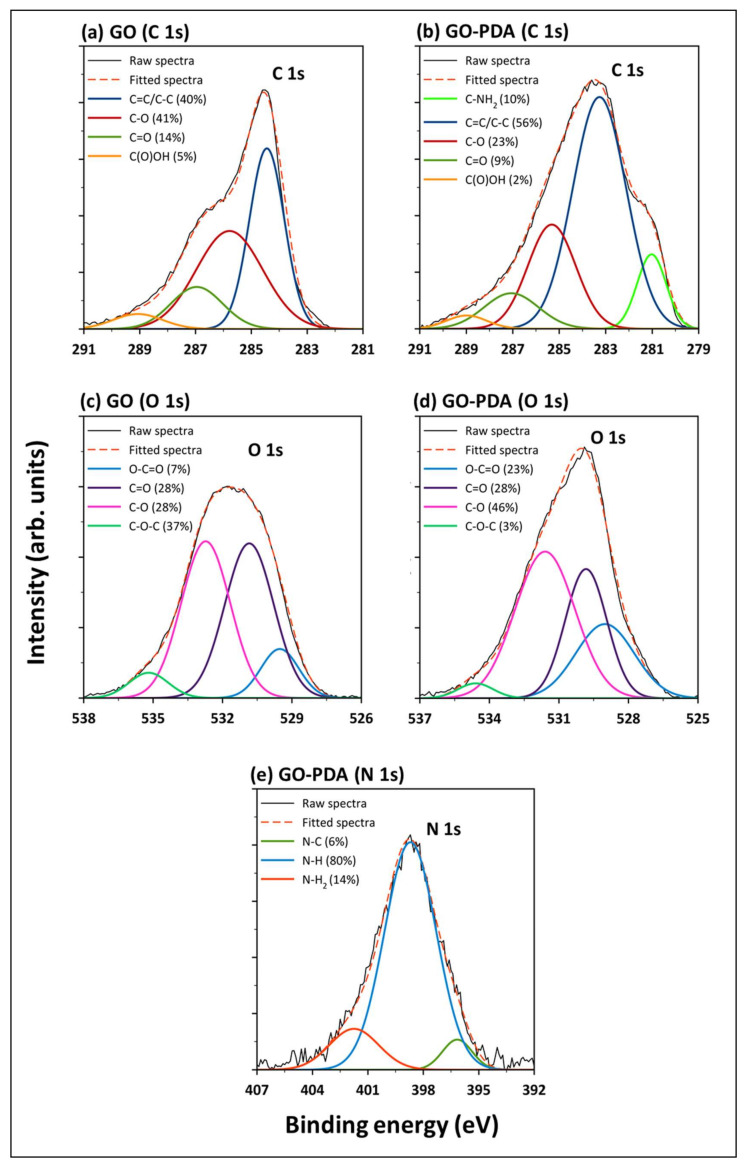
High-resolution and deconvolution of XPS spectra of (**a**) GO C 1s, (**b**) GO-PDA C 1s, (**c**) GO O 1s, (**d**) GO-PDA O 1s, and (**e**) GO-PDA N 1s core-levels.

XPS survey spectra were recorded to explore the surface elemental compositions, nature, and the chemical states of the functional groups of both samples. The complete XPS survey spectra of GO and GO-PDA in Figure 9 shows the existence of O 1s and C 1s core-levels around binding energies of 531 and 284 eV, respectively. The small peaks at 166 eV correspond to S 2p indicating the presence of trace amounts of sulphur in both samples. The N 1s peak emerged in GO-PDA spectra at binding energy of ~398 eV indicates that GO was successfully aminated with PDA. Additional two peaks emerged in the spectra of GO-PDA around 196 and 266 eV corresponding to Cl 2p and Cl 2s due to the chlorine content of dopamine. For a better interpretation of the XPS spectra, C 1s and O 1s core-levels were deconvoluted and the abundance percentage of functional groups are presented in Figure 10a–d. Four peaks were obtained by deconvoluting the C 1 s region of GO: C=C/C–C (40%) at a binding energy of 284 eV attributed to sp^2^ bound graphitic carbon, C–O (41%) at 286 eV related to the epoxide groups, C=O (14%) of the carbonyl functional groups at 287 eV, and the carboxylic group on the GO edges (COOH) at 289 eV (5%). These peaks were reported in the literature for GO NPs [5,33,51]. Similar peaks were obtained from the deconvolution of C 1s core-level of GO-PDA in addition to a fifth peak corresponding to C–NH_2_ at a binding energy of 281 eV emerged from the PDA amine groups [52], which resulted in a slight reduction in the compositions of oxygen-containing groups. The deconvolution of the O 1s spectra illustrated in Figure 10c,d resulted into four main peaks, O–C=O, C=O, C–O, and C–O–C, at binding energies of ~530, 531, 533, and 535 eV, respectively [4]. The N 1s peak emerged in the GO-PDA spectra was deconvoluted and fitted into three main peaks at binding energies of 402, 399, and 396 eV corresponding to primary amine (N–H_2_), secondary amine (N–H), and aromatic/tertiary amine (N–C) functional groups, respectively, as illustrated in Figure 10e [53,54,55]. The primary amine is linked to the dopamine, the secondary amine corresponds to the polydopamine and the tertiary amine corresponds to the tautomers of 5,6-dihydroxyindole and 5,6-indolequinone [54]. Peak parameters and the corresponding atomic compositions estimated from the XPS fit are listed in Table S3 of the supplementary information.

Table 3 and Table 4 list the elemental compositions of GO and GO-PDA as determined by the XPS and CHNSO elemental analyzer, respectively. When comparing the compositions obtained by the two techniques, it is clearly seen that the carbon is overestimated in the XPS analysis for both samples which is previously observed in some studies in the literature [4,5,33,56]. The difference between the elemental compositions obtained from the two techniques can be related to the fact that the CHNSO analyzer gives information about the bulk sample [57,58]; while XPS can only provide information about the average distribution on the surface only [59]. The results obtained from CHNSO analysis show a good oxidation degree of graphite to GO with an oxygen content of 50 wt. % and an atomic O/C ratio of 0.8. However, the functionalization with PDA resulted in partial reduction of GO lowering its oxygen content to 37.7 wt. % and O/C ratio to 0.6 which agrees with the FTIR results. The reduction is not as significant as this reported with other amines like n-butylamine [11], dodecylamine [60] or melamine [61], which can be ascribed to the oxygenated functional groups of PDA that compensated some of the reduced oxygen in GO.

### 3.3. Dispersibility and Hydrophilicity

**Figure 11:**
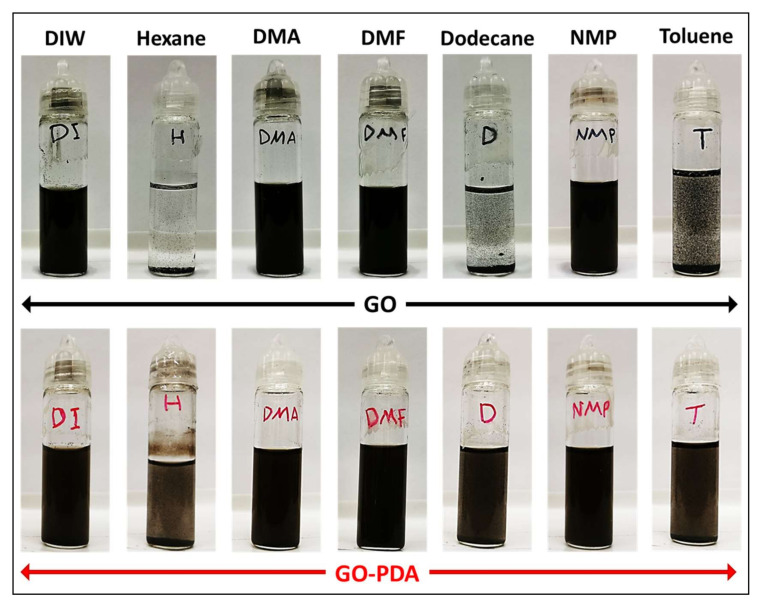
Photographs of GO and GO-PDA dispersions in various solvents.

**Figure 12:**
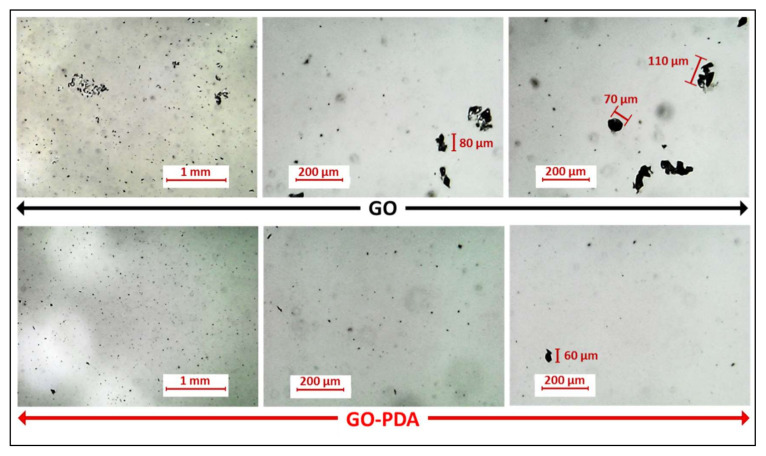
Optical micrographs of GO and GO-PDA dispersion in DIW (0.02 mg/mL).

Figure 11 presents photographs of GO and GO-PDA suspensions after sonication. Pristine GO exhibited good dispersibility in DIW, NMP, DMF, and DMA; while with other solvents (i.e., hexane, toluene, and dodecane), the dispersibility was very poor. These observations were previously reported in some studies in literature and are mainly attributed to the polarity of GO [11,62]. In contrast, GO-PDA showed high dispersion in all solvents except in hexane with average dispersibility. The high dispersibility of GO-PDA in polar and nonpolar solvents expands its potential uses for various applications over the pristine GO including surface functionalization of RO and NF membranes [8,63], anticorrosive coatings [64], conductive inks [65], and oil recovery [66]. Figure 12 presents the optical micrographs of GO and GO-PDA dispersion in DIW with a low concentration (0.02 mg/mL). It is obvious that the pristine GO NPs agglomerate even at this low concentration. In contrast, GO-PDA NPs showed less agglomeration and have better dispersibility in water than the pristine GO. This observation indicates that GO-PDA NPs have higher dispersion stability in water with less agglomeration. This could be attributed to the hydrophilic functional groups of PDA that provide better dispersibility and stability of GO NPs.

**Figure 13:**
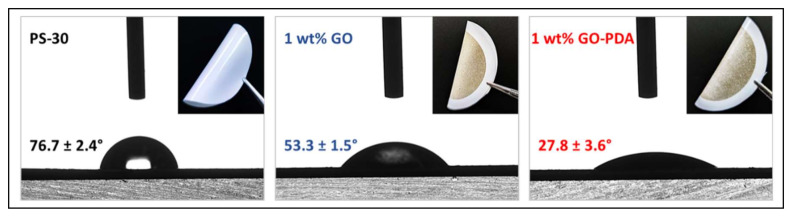
Contact angle of PS-30, PS/GO, and PS/GO-PDA membranes.

Figure 13 depicts photographs of the pristine PS-30 and the NPs coated membranes with their hydrophilicity in terms of contact angle. Obviously, the surface hydrophilicity of PS-30 was slightly enhanced with GO NPs deposition. The average contact angle of the pristine PS-30 substrate was measured to be 76.7° while it was reduced to 53.3° after the coating with the GO layer. This observation has been reported in several studies with different membrane materials and was attributed to the hydrophilic nature of GO [67,68]. Interestingly, the contact angle was much lower (27.8°) with the assembly of the GO-PDA layer suggesting higher hydrophilicity of GO-PDA than the pristine GO NPs. The high hydrophilicity of GO-PDA can be explained by the presence of abundant hydroxyl groups of PDA that is consistent with the predicted chemical structure of GO-PDA NPs in Figure 2. The high hydrophilicity accompanied by the high dispersibility makes the GO-PDA NPs efficient nanofiller in membrane technology. The high dispersibility allows the utilization of GO-PDA with different types of membranes that use NMP, DMF, and DMA to prepare mixed matrix membranes (MMMs), dodecane or hexane, to prepare thin-film nanocomposite (TFN) membranes, or water to prepare coated membranes based on assembly approach. In a recent study, we compared the effect of embedding pristine GO NPs and GO-PDA on the ultrafiltration performance of polysulfone (PSF) mixed matrix membranes (MMMs) [69]. Different concentrations of the pristine GO and GO-PDA NPs were embedded into the PSF matrix using the well-known phase inversion technique. The results showed superior enhancements in the flux without influencing the rejection. PSF MMMs incorporating 0.1 wt. % GO-PDA exhibited a pure water permeability of 326.5 LMH/bar compared to 182.9 and 164.5 LMH/bar with pristine PSF and PSF incorporating the same concentration of pristine GO. Additionally, GO-PDA-based membranes showed high antifouling performance against organic and protein fouling and exhibited 30% increase in the flux recovery ration (FRR) compared to the pristine PSF. The advantageous performance of GO-PDA membranes is mainly attributed to the higher dispersibility and hydrophilicity of the functionalized GO compared to the pristine GO NPs. One of the main drawbacks of pristine GO is the poor dispersion in some solvents which causes agglomeration and hence limiting the overall performance of the membrane [68,70]. The use of GO-PDA NPs is not limited to MMMs as the high dispersibility would enhance their utilization with different membrane processes and with different materials.

### 3.4. Thermal Stability

**Figure 14:**
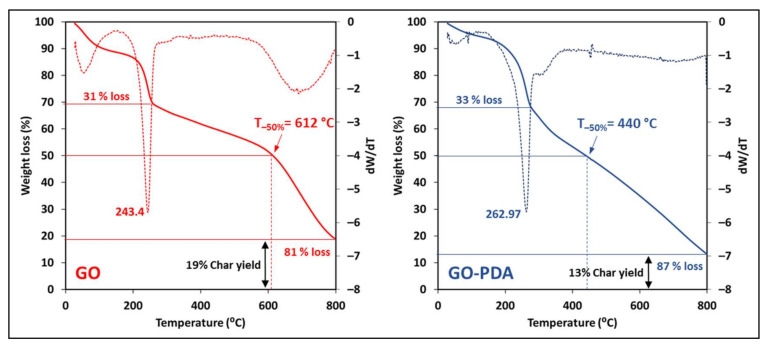
TGA curves and the corresponding derivative curves of GO and GO-PDA.

The thermal stability of the pristine GO and GO-PDA was studied using TGA analysis. Figure 14 illustrates the TGA curves of GO and GO-PDA and their corresponding derivatives. Three stages of weight loss can be observed from the TGA curves of both samples. A minor weight loss around 100 °C attributed to the release of water intercalated between GO sheets [6,71]. The second stage represents the thermal decomposition of unstable oxygenic functional groups (carboxyl, epoxy, and hydroxyl) [33,72] resulting in a major weight loss of the sample. The major weight loss of GO and GO-PDA occurred around 243 and 263 °C, respectively, as depicted by the derivative curves. This indicates better thermal stability, at this stage, of GO-PDA than the pristine GO which can be explained by the partial GO reduction and the replacement of some oxygen-containing functional groups with amine groups that slow down the thermal decomposition. The final stage of thermal decomposition occurs then at high temperatures at which the most stable functional groups decompose [33,38]. The weight of GO-PDA at the final stage decreased sharply to 50% around 440 °C; while the 50% loss occurred around 612 °C for pristine GO. The TGA curves also suggest a lower char yield of GO-PDA (about 35% at 600 °C and 13% at 800 °C) compared to the pristine GO (about 52% at 600 °C and 19% at 800 °C). The difference in thermal stability of GO and GO-PDA can be related to the different functional groups presented in each sample, as the thermal decomposition is highly dependent on the bond dissociation energies [33].

## 4. Conclusions

Facile functionalization of GO NPs with PDA has been reported in this work to obtain highly aminated GO NPs with superior hydrophilic properties. Starting from natural graphite, GO NPs were synthesized using a modified Hummers method. All characterization techniques confirmed the successful functionalization of GO NPs. FTIR-UATR and XPS spectra showed the presence of several nitrogen-containing functional groups corresponding to amine groups of PDA. Also, a partial reduction in oxygen content was confirmed by XRD, FTIR-UATR, Raman spectra, XPS, and CHNSO elemental analysis caused by the reduction of C=O in functionalized GO. Raman spectra showed the two typical bands of GO (D and G) in both samples with a lower *I_D_*/*I_G_* ratio for GO-PDA suggesting different crystallite properties and defects and lower oxygen content. This was also confirmed by XRD analysis which showed different interlayer spacing (d-spacing) for both samples. The interlayer spacing for GO-PDA was higher than this of pristine GO that can be attributed to the presence of newly formed oxygenic functional groups from PDA. TGA analysis showed that GO-PDA decomposes faster and has a lower char yield than the pristine GO due to the different functional groups in each sample. Interestingly, GO-PDA showed better dispersibility in polar solvents (water, DMF, DMA, and NMP) and nonpolar solvents (hexane, dodecane, and toluene). The high dispersibility expands the range of potential applications of GO-PDA. Further, the hydrophilicity represented by contact angle was significantly improved because of the abundant hydroxyl groups of PDA, even with low concentrations of GO-PDA layer (1 wt. %) on the PS substrate. Therefore, GO-PDA has the potential as nanofiller for modifying different membrane materials and types due to the higher hydrophilicity and dispersibility.

## Figures and Tables

**Table 1:** Recently reported amine-functionalized graphene oxide (GO) materials and their applications.

Aminated-GO	Application	Ref.
GO-p-aminophenol	RO desalination	[8]
GO-ODA	NF desalination	[9]
GO-TEOA ^a^	NF desalination	[17]
GO-APTS	UF (Natural organic matter removal)	[18]
GO-ButA	Membrane distillation (MD)	[10]
GO-MXDA ^b^	FO desalination	[19]

^a^ TEOA: Triethanolamine; ^b^ MXDA: m-xylylenediamine.

**Table 2:** D and G bands’ parameters of Raman spectra and the estimated crystallite size.

GO Sample	Curve	Peak Center (cm^−1^)	Peak Area (arb. unit)	*I_D_*/*I_G_*	*La*
GO	D	1352	334053	1.8	10.9
	G	1588	189857		
GO-PDA	D	1352	59039	1.3	15.2
	G	1583	46638		

**Table 3:** XPS elemental compositions of the GO and GO-PDA NPs.

Sample	Atomic Composition (at.%)					
	C 1s	O 1s	N 1s	S 2p	Cl 2p	Cl 2s
GO	74.58	24.52	0.5	0.4	-	-
GO-PDA	69.94	20.61	6.61	0.11	1.82	0.91

**Table 4:** CHNSO elemental compositions of the GO and GO-PDA NPs.

Sample	Wt.%					At.%					
	N	C	H	S	O	N	C	H	S	O	O/C
GO	0.3	46.8	2.6	0.3	50.0	0.2	40.3	27.1	0.1	32.3	0.80
GO-PDA	7.8	49.8	4.8	0.0	37.7	4.7	35.2	40.1	0.0	20.0	0.6

## Supplementary Materials

The following are available online at https://www.mdpi.com/2077-0375/11/2/86/s1, Table S1: Bands parameters estimated from the Raman first-order spectra fits, Table S2: Bands parameters estimated from the Raman second-order spectra fits, Table S3: Peaks parameters and the atomic compositions estimated from the XPS spectra fits.

## References

[1] Stankovich S., Dikin D.A., Dommett G.H.B., Kohlhaas K.M., Zimney E.J., Stach E.A., Piner R.D., Nguyen S.T., Ruoff R.S. (2006). Graphene-based composite materials. Nat. Cell Biol..

[2] Hummers W.S., Offeman R.E. (1958). Preparation of Graphitic Oxide. J. Am. Chem. Soc..

[3] Lerf A., He H., Forster M., Klinowski J. (1998). Structure of Graphite Oxide Revisited. J. Phys. Chem. B.

[4] Al-Gaashani R., Najjar A., Zakaria Y., Mansour S., Atieh M. (2019). XPS and structural studies of high quality graphene oxide and reduced graphene oxide prepared by different chemical oxidation methods. Ceram. Int..

[5] Muzyka R., Kwoka M., Smędowski Ł., Díez N., Gryglewicz G. (2017). Oxidation of graphite by different modified Hummers methods. New Carbon Mater..

[6] Chen J., Li Y., Huang L., Li C., Shi G. (2015). High-yield preparation of graphene oxide from small graphite flakes via an improved Hummers method with a simple purification process. Carbon.

[7] Ashfaq M.Y., Al-Ghouti M.A., Da’Na D.A., Qiblawey H., Zouari N. (2020). Investigating the effect of temperature on calcium sulfate scaling of reverse osmosis membranes using FTIR, SEM-EDX and multivariate analysis. Sci. Total Environ..

[8] Zhang Y., Ruan H., Guo C., Liao J., Shen J., Gao C. (2020). Thin-film nanocomposite reverse osmosis membranes with enhanced antibacterial resistance by incorporating p-aminophenol-modified graphene oxide. Sep. Purif. Technol..

[9] Xue S.-M., Ji C.-H., Xu Z.-L., Tang Y.-J., Li R.-H. (2018). Chlorine resistant TFN nanofiltration membrane incorporated with octadecylamine-grafted GO and fluorine-containing monomer. J. Membr. Sci..

[10] Lu K.-J., Zuo J., Chung T.-S. (2017). Novel PVDF membranes comprising n-butylamine functionalized graphene oxide for direct contact membrane distillation. J. Membr. Sci..

[11] Chakraborty S., Saha S., Dhanak V.R., Biswas K., Barbezat M., Terrasi G.P., Chakraborty A.K. (2016). High yield synthesis of amine functionalized graphene oxide and its surface properties. RSC Adv..

[12] Song M.G., Choi J., Jeong H.E., Song K., Jeon S., Cha J., Baeck S.-H., Shim S.E., Qian Y. (2020). A comprehensive study of various amine-functionalized graphene oxides for room temperature formaldehyde gas detection: Experimental and theoretical approaches. Appl. Surf. Sci..

[13] Ramesh P., Bhagavathsingh J. (2019). A facile synthesis of bis-(pththalimidoethyl)-amine functionalized graphene oxide and its dual performance as a supercapacitor electrode and fluorescence sensor. Mater. Chem. Phys..

[14] Rana S., Jonnalagadda S.B. (2017). Cu doped amine functionalized graphene oxide and its scope as catalyst for selective oxidation. Catal. Commun..

[15] Liu Y., Sajjadi B., Egiebor N.O., Chatterjee R. (2019). Ultrasound-assisted amine functionalized graphene oxide for enhanced CO_2_ adsorption. Fuel.

[16] Ebrahimi-Tazangi F., Hekmatara H., Seyed-Yazdi J., Hekmatara H. (2019). Synthesis and remarkable microwave absorption properties of amine-functionalized magnetite/graphene oxide nanocomposites. J. Alloys Compd..

[17] Nakagawa K., Araya S., Kunimatsu M., Yoshioka T., Shintani T., Kamio E., Matsuyama H. (2018). Fabrication of Stacked Graphene Oxide Nanosheet Membranes Using Triethanolamine as a Crosslinker and Mild Reducing Agent for Water Treatment. Membranes.

[18] Kumar M., Sreedhar N., Jaoude M.A., Arafat H.A. (2019). High-Flux, Antifouling Hydrophilized Ultrafiltration Membranes with Tunable Charge Density Combining Sulfonated Poly(ether sulfone) and Aminated Graphene Oxide Nanohybrid. ACS Appl. Mater. Interfaces.

[19] Jin L., Wang Z., Zheng S., Mi B. (2018). Polyamide-crosslinked graphene oxide membrane for forward osmosis. J. Membr. Sci..

[20] Lee H., Dellatore S.M., Miller W.M., Messersmith P.B. (2007). Mussel-Inspired Surface Chemistry for Multifunctional Coatings. Science.

[21] Wang T., Qiblawey H., Judd S., Benamor A., Nasser M., Mohammadian M. (2018). Fabrication of high flux nanofiltration membrane via hydrogen bonding based co-deposition of polydopamine with poly(vinyl alcohol). J. Membr. Sci..

[22] Wang T., Qiblawey H., Sivaniah E., Mohammadian A. (2016). Novel methodology for facile fabrication of nanofiltration membranes based on nucleophilic nature of polydopamine. J. Membr. Sci..

[23] Sun R., Renb F., Wang N., Yao Y., Fei Z., Wang H., Liu Z., Xing R., Du Y. (2019). Polydopamine functionalized multi-walled carbon nanotubes supported PdAu nanoparticles as advanced catalysts for ethylene glycol oxidation. Colloids Surf. A Physicochem. Eng. Asp..

[24] Kumar A., Mishra B., Tripathi B.P. (2020). Polydopamine assisted synthesis of ultrafine silver nanoparticles for heterogeneous catalysis and water remediation. Nano-Struct. Nano-Objects.

[25] Cui J., Zhou Z., Xie A., Meng M., Cui Y., Liu S., Lu J., Zhou S., Yan Y., Dong H. (2019). Bio-inspired fabrication of superhydrophilic nanocomposite membrane based on surface modification of SiO_2_ anchored by polydopamine towards effective oil-water emulsions separation. Sep. Purif. Technol..

[26] Sun X., Yan L., Xu R., Xu M., Zhu Y. (2019). Surface modification of TiO_2_ with polydopamine and its effect on photocatalytic degradation mechanism. Colloids Surf. A Physicochem. Eng. Asp..

[27] Zhao Z., Guo L., Feng L., Lu H., Xu Y., Wang J., Xiang B., Zou X. (2019). Polydopamine functionalized graphene oxide nanocomposites reinforced the corrosion protection and adhesion properties of waterborne polyurethane coatings. Eur. Polym. J..

[28] Faraji M., Gharibi H., Javaheri M. (2016). High Pt Loading on Polydopamine Functionalized Graphene as a High Performance Cathode Electrocatalyst for Proton Exchange Membrane Fuel Cells. J. Nanostruct..

[29] Palanisamy S., Thirumalraj B., Chen S.-M., Wang Y.-T., Velusamy V., Ramaraj S.K. (2016). A Facile Electrochemical Preparation of Reduced Graphene Oxide@Polydopamine Composite: A Novel Electrochemical Sensing Platform for Amperometric Detection of Chlorpromazine. Sci. Rep..

[30] Alkhouzaam A., Qiblawey H., Khraisheh M., Atieh M., Al-Ghouti M. (2020). Synthesis of graphene oxides particle of high oxidation degree using a modified Hummers method. Ceram. Int..

[31] Shakeri A., Salehi H., Rastgar M. (2019). Antifouling electrically conductive membrane for forward osmosis prepared by polyaniline/graphene nanocomposite. J. Water Process. Eng..

[32] Panicker N.J., Das J., Sahu P. (2020). Synthesis of highly oxidized graphene (HOG) by using HNO_3_ and KMnO_4_ as oxidizing agents. Mater. Today Proc..

[33] Yadav N., Lochab B. (2019). A comparative study of graphene oxide: Hummers, intermediate and improved method. FlatChem.

[34] Manwatkar H., Gedam S., Bhaskar C., Dhonde M., Thakare S.R. (2020). Synthesis and properties of amino and thiol functionalized graphene oxide. Mater. Today Proc..

[35] Xu L.Q., Yang W.J., Neoh K.-G., Kang E.-T., Fu G. (2010). Dopamine-Induced Reduction and Functionalization of Graphene Oxide Nanosheets. Macromolecules.

[36] Lee M., Ku S.H., Ryu J., Park C.B. (2010). Mussel-inspired functionalization of carbon nanotubes for hydroxyapatite mineralization. J. Mater. Chem..

[37] Liu Y., Tu W., Chen M., Ma L., Yang B., Liang Q., Chen Y. (2018). A mussel-induced method to fabricate reduced graphene oxide/halloysite nanotubes membranes for multifunctional applications in water purification and oil/water separation. Chem. Eng. J..

[38] Marcano D.C., Kosynkin D.V., Berlin J.M., Sinitskii A., Sun Z., Slesarev A., Alemany L.B., Lu W., Tour J.M. (2010). Improved Synthesis of Graphene Oxide. ACS Nano.

[39] Guerrero-Contreras J., Caballero-Briones F. (2015). Graphene oxide powders with different oxidation degree, prepared by synthesis variations of the Hummers method. Mater. Chem. Phys..

[40] Ma Y.-R., Zhang X., Zeng T., Cao D., Zhou Z., Li W.-H., Niu H., Cai Y. (2013). Polydopamine-Coated Magnetic Nanoparticles for Enrichment and Direct Detection of Small Molecule Pollutants Coupled with MALDI-TOF-MS. ACS Appl. Mater. Interfaces.

[41] Coskun H., Aljabour A., Uiberlacker L., Strobel M., Hild S., Cobet C., Farka D., Stadler P., Sariciftci N.S. (2018). Chemical vapor deposition-based synthesis of conductive polydopamine thin-films. Thin Solid Films.

[42] Wang J., Zhou S., Huang J., Zhao G., Liu Y., Wang J., Zhou S., Huang J., Zhao G., Liu Y. (2018). Interfacial modification of basalt fiber filling composites with graphene oxide and polydopamine for enhanced mechanical and tribological properties. RSC Adv..

[43] Malard L.M., Pimenta M.A., Dresselhaus G., Dresselhaus M.S. (2009). Raman spectroscopy in graphene. Phys. Rep..

[44] Lowe S.E., Shi G., Zhang Y., Qin J., Jiang L., Jiang S., Al-Mamun M., Liu P., Zhong Y.L., Zhao H. (2019). The role of electrolyte acid concentration in the electrochemical exfoliation of graphite: Mechanism and synthesis of electrochemical graphene oxide. Nano Mater. Sci..

[45] Claramunt S., Varea A., López-Díaz D., Velázquez M.M., Cornet A., Cirera A. (2015). The Importance of Interbands on the Interpretation of the Raman Spectrum of Graphene Oxide. J. Phys. Chem. C.

[46] Sadezky A., Muckenhuber H., Grothe H., Niessner R., Pöschl U. (2005). Raman microspectroscopy of soot and related carbonaceous materials: Spectral analysis and structural information. Carbon.

[47] López-Díaz D., Holgado M.L., García-Fierro J.L., Velázquez M.M. (2017). Evolution of the Raman Spectrum with the Chemical Composition of Graphene Oxide. J. Phys. Chem. C.

[48] Tuinstra F., Koenig J.L. (1970). Raman Spectrum of Graphite. J. Chem. Phys..

[49] Ferrari A.C., Meyer J.C., Scardaci V., Casiraghi C., Lazzeri M., Mauri F., Piscanec S., Jiang D., Novoselov K.S., Roth S. (2006). Raman Spectrum of Graphene and Graphene Layers. Phys. Rev. Lett..

[50] Zhu Y., Murali S., Cai W., Li X., Suk J.W., Potts J.R., Ruoff R.S. (2010). Graphene and Graphene Oxide: Synthesis, Properties, and Applications. Adv. Mater..

[51] Krishnamoorthy K., Veerapandian M., Yun K., Kim S.-J. (2013). The chemical and structural analysis of graphene oxide with different degrees of oxidation. Carbon.

[52] Patel K., Singh N., Yadav J., Nayak J.M., Sahoo S.K., Lata J., Chand D., Kumar S., Kumar R. (2018). Polydopamine films change their physicochemical and antimicrobial properties with a change in reaction conditions. Phys. Chem. Chem. Phys..

[53] Liu S., Qileng A., Huang J., Gao Q., Liu Y. (2017). Polydopamine as a bridge to decorate monodisperse gold nanoparticles on Fe3O4 nanoclusters for the catalytic reduction of 4-nitrophenol. RSC Adv..

[54] Liu T., Kim K.C., Lee B., Chen Z., Noda S., Jang S.S., Lee S.W. (2017). Self-polymerized dopamine as an organic cathode for Li- and Na-ion batteries. Energy Environ. Sci..

[55] Li X., Shan H., Cao M., Li B., Xipeng L., Huiting S., Min C., Baoan L. (2018). Mussel-inspired modification of PTFE membranes in a miscible THF-Tris buffer mixture for oil-in-water emulsions separation. J. Membr. Sci..

[56] Tobi A.R., Dennis J., Zaid H., Adekoya A., Yar A., Fahad U. (2019). Comparative analysis of physiochemical properties of physically activated carbon from palm bio-waste. J. Mater. Res. Technol..

[57] Plata D.L., Gschwend P.M., Reddy C.M. (2008). Industrially synthesized single-walled carbon nanotubes: Compositional data for users, environmental risk assessments, and source apportionment. Nanotechnology.

[58] Braun E.I., Pantano P. (2014). The importance of an extensive elemental analysis of single-walled carbon nanotube soot. Carbon.

[59] Pumera M. (2007). Carbon Nanotubes Contain Residual Metal Catalyst Nanoparticles even after Washing with Nitric Acid at Elevated Temperature Because These Metal Nanoparticles Are Sheathed by Several Graphene Sheets. Langmuir.

[60] Ren P.-G., Wang H., Huang H.-D., Yan D.-X., Li Z.-M. (2013). Characterization and performance of dodecyl amine functionalized graphene oxide and dodecyl amine functionalized graphene/high-density polyethylene nanocomposites: A comparative study. J. Appl. Polym. Sci..

[61] Ajdari F.B., Kowsari E., Ehsani A., Chepyga L., Schirowski M., Jäger S., Kasian O., Hauke F., Ameri T. (2018). Melamine-functionalized graphene oxide: Synthesis, characterization and considering as pseudocapacitor electrode material with intermixed POAP polymer. Appl. Surf. Sci..

[62] Du W., Wu H., Chen H., Xu G., Li C. (2020). Graphene oxide in aqueous and nonaqueous media: Dispersion behaviour and solution chemistry. Carbon.

[63] Shao W., Liu C., Ma H., Hong Z., Xie Q., Lu Y. (2019). Fabrication of pH-sensitive thin-film nanocomposite nanofiltration membranes with enhanced performance by incorporating amine-functionalized graphene oxide. Appl. Surf. Sci..

[64] Jing L.-C., Wang T., Cao W.-W., Wen J.-G., Zhao H., Ning Y.-J., Yuan X.-T., Tian Y., Teng L.-H., Geng H. (2020). Water-based polyurethane composite anticorrosive barrier coating via enhanced dispersion of functionalized graphene oxide in the presence of acidified multi-walled carbon nanotubes. Prog. Org. Coat..

[65] Konios D., Stylianakis M.M., Stratakis E., Kymakis E. (2014). Dispersion behaviour of graphene oxide and reduced graphene oxide. J. Colloid Interface Sci..

[66] Aliabadian E., Sadeghi S., Moghaddam A.R., Maini B.B., Chen Z., Sundararaj U. (2020). Application of graphene oxide nanosheets and HPAM aqueous dispersion for improving heavy oil recovery: Effect of localized functionalization. Fuel.

[67] Chang X., Wang Z., Quan S., Xu Y., Jiang Z., Shao L. (2014). Exploring the synergetic effects of graphene oxide (GO) and polyvinylpyrrodione (PVP) on poly(vinylylidenefluoride) (PVDF) ultrafiltration membrane performance. Appl. Surf. Sci..

[68] Jiang Y., Zeng Q., Biswas P., Fortner J. (2019). Graphene oxides as nanofillers in polysulfone ultrafiltration membranes: Shape matters. J. Membr. Sci..

[69] Alkhouzaam A., Qiblawey H. (2020). Novel polysulfone ultrafiltration membranes incorporating polydopamine functionalized graphene oxide with enhanced flux and fouling resistance. J. Membr. Sci..

[70] Alkhouzaam A., Qiblawey H., Khraisheh M. (2020). Synthesis of High-Antifouling and Antibacterial Ultrafiltration Membranes Incorporating Low Concentrations of Graphene Oxide. Qatar University Annual Research Forum and Exhibition (QUARFE 2020).

[71] Naeem H., Ajmal M., Qureshi R.B., Muntha S.T., Farooq M., Siddiq M. (2019). Facile synthesis of graphene oxide–silver nanocomposite for decontamination of water from multiple pollutants by adsorption, catalysis and antibacterial activity. J. Environ. Manag..

[72] Talyzin A.V., Mercier G., Klechikov A., Hedenström M., Johnels D., Wei D., Cotton D., Opitz A., Moons E. (2017). Brodie vs Hummers graphite oxides for preparation of multi-layered materials. Carbon.

