# Supplementary material for: Polydopamine Functionalized Graphene Oxide as Membrane Nanofiller: Spectral and Structural Studies
Source: Membranes (Basel). 2021 Jan 27;11(2):86. doi: 10.3390/membranes11020086 (PMC7910935; doi:10.3390/membranes11020086)
Supplement: Supplementary file 1 [file membranes-11-00086-s001.pdf]

membranes

Article

Polydopamine Functionalized Graphene Oxide as Membrane Nanofiller: Spectral and Structural Studies

Abedalkader Alkhouzaam, Hazim Qiblawey * and Majeda Khraisheh

Department of Chemical Engineering, College of Engineering, Qatar University, P. O. Box 2713, Doha, Qatar; 200602139@student.qu.edu.qa (A.A.); m.khraisheh@qu.edu.qa (M.K.)
* Correspondence: hazim@qu.edu.qa

Received: 5 November 2020; Accepted: 17 December 2020 Published: date

Table S1: Bands parameters estimated from the Raman first-order spectra fits

Sample	Band	Raman shift (cm^{-1})	Peak intensity (arb. units)	Peak area (arb. units)
GO	D	1352	1737	334053
	D''	1496	282	55642
	G	1588	1736	189858
	D'	1619	394	22390
GO-PDA	D	1352	282	59039
	D''	1492	72	8735
	G	1583	292	46638
	D'	1603	66	2724
	D*	1188	53	9783

Table S2: Bands parameters estimated from the Raman second-order spectra fits

Sample	Band	Raman shift (cm^{-1})	Peak intensity (arb. units)	Peak area (arb. units)
GO	2D	2685	199	28497
	D+D'	2938	353	46929
	2D'	3193	118	9796
GO-PDA	2D	2722	69	31083
	D+D'	2950	56	12224
	2D'	3160	51	12439

Table S3: Peaks parameters and the atomic compositions estimated from the XPS spectra fits.

Sample	Peak	functional group	Binding energy (eV)	Peak area (arb. units)	at. %
GO	C 1s	C-C	284.4	10026	11.48
	C 1s	C-O	285.8	10334	11.84
	C 1s	C=O	287.0	3444	3.94
	C 1s	C(O)OH	289.0	1170	1.34
	O 1s	O-C=O	529.5	4284	4.91
	O 1s	C=O	530.8	17439	19.97
	O 1s	C-O	532.7	17757	20.34
	O 1s	C-O-C	535.2	22856	26.18
GO-PDA	C 1s	$C-NH_2$	281.0	4129	4.36
	C 1s	C-C	283.3	23635	24.98
	C 1s	C-O	285.3	9495	10.04
	C 1s	C=O	287.0	3684	3.89
	C 1s	C(O)OH	289.0	1020	1.08
	O 1s	O-C=O	529.0	9978	10.55
	O 1s	C=O	529.8	12369	13.07
	O 1s	C-O	531.6	20223	21.37
	O 1s	C-O-C	534.6	1202	1.27
	N 1s	N-C	396.2	538	0.57
	N 1s	N-H	398.7	7125	7.53
	N 1s	$N-H^+$	401.8	1216	1.29
